# Diphtheria, Muriate of Pilocarpine

**Published:** 1881-02

**Authors:** 


DIPHTHERIA. MURIATE OF PILOCARPINE has been recommended in the highest terms by Dr. Guttmann, of Constadt, and has been endorsed by Lax. in an unequivocal manner. If the statements of those observers should be confirmed by the experience of others, a specific has been found against one of the most dreaded and often intractable diseases which the physician is called upon to encounter. Guttmann's plan is as follows:

He administers the drug in conjunction with pepsine, since the latter may, as he says, have a solvent effect upon the diphtheritic membrane. In adults half a grain, to one grain, of the muriate of pilocarpine is exhibited in an eight ounce mixture. In addition to this ingredient, the solution also contains half a drachm of pepsine, and a few drops of muriatic acid. Adult patients are directed to take the medicine hourly in tablespoonful doses. As a necessary adjuvant towards a proper action of the remedy, the use of strong wine (half an ounce at a time, after each dose of the medicine) is insisted upon, Children are required to take proportionately less, both of the drug and the wine.

In no case did Guttmann observe unpleasant symptoms follow the use of the alkaloid. Its sialogogue action was always promptly manifested. With the establishment of copious expectoration the febrile movement diminished in intensity, and the local symptoms rapidly improved. The patients are said to have been cured in from one to four days. He observed recoveries under this regimen of patients who, under ordinary plans of treatment, must have been considered as hopeless cases.

